# Absolute iodine concentration for dynamic perfusion imaging of the myocardium: improved detection of poststenotic ischaemic in a 3D-printed dynamic heart phantom

**DOI:** 10.1186/s41747-022-00304-x

**Published:** 2022-10-31

**Authors:** Johannes Hammel, Lorenz Birnbacher, Marcus R. Makowski, Franz Pfeiffer, Daniela Pfeiffer

**Affiliations:** 1 Department of Diagnostic and Interventional Radiology, School of Medicine, Klinikum rechts der Isar, Technical University of Munich, Klinikum rechts der Isar, Ismaninger Str. 22, 81675 Munich, Germany; 2 Chair of Biomedical Physics, Department of Physics, School of Natural Sciences, Technical University of Munich, Garching, Germany; 3 Munich Institute of Biomedical Engineering, Technical University of Munich, Garching, Germany; 4 Institute for Advanced Study, Technical University of Munich, Garching, Germany

**Keywords:** Coronary stenosis, Myocardial ischemia, Perfusion imaging, Printing (three-dimensional), Tomography (x-ray computed)

## Abstract

**Background:**

To investigate the detection capabilities of myocardial perfusion defects of dual-energy computed tomography (CT) technology using time-resolved iodine-based maps for functional assessment of coronary stenosis in a dynamic heart phantom.

**Methods:**

An anatomical heart model was designed using a three-dimensional (3D) printing technique. The lumen of the right coronary artery was reduced to 25% of the original areal cross-section. Scans were acquired with a 64-slice dual-layer CT equipment using a perfusion protocol with 36 time points. For distinguishing haemodynamically affected from unaffected myocardial regions, conventional and spectral mean transit time (MTT) parameter maps were compared. A dose reduction technique was simulated by using a subset of time points of the time attenuation curves (TACs).

**Results:**

The tracer kinetic modeling showed decreased errors on fit parameters from conventional to spectral TACs (42% reduction for A and 40% for λ). Three characteristic regions (highly, moderately, and not affected by the simulated stenosis) can be distinguished in all spectral perfusion maps. The best distinction was observed on MTT maps. An area under the curve (AUC) value of 1.00 for the voxel-wise differentiation of haemodynamically affected tissue was achieved *versus* a 0.89 AUC for conventional MTT maps. By temporal under-sampling, a dose reduction of approximately 78% from 19 to 4.3 mSv was achieved with a 0.96 AUC.

**Conclusion:**

Dual-energy CT can provide time-resolved iodine density data, which enables the calculation of absolute quantitative perfusion maps with decreased fitting errors, improving the accuracy for poststenotic myocardial ischaemic detection in a 3D-printed heart phantom.

**Supplementary Information:**

The online version contains supplementary material available at 10.1186/s41747-022-00304-x.

## Key points

- Increased fit accuracy on spectral computed tomography perfusion data compared to the conventional technique.
- Possibility of voxel-wise differentiation between ischaemic and nonischaemic myocardial tissue.
- Implementation of dose reduction technique by temporal under-sampling.

## Background

Along with major advances in cardiovascular science and medicine, a steady decline in deaths from cardiovascular disease in relation to scientific advances can be observed [1, 2]. Anatomical visualisation of coronary artery disease (CAD) can be guaranteed by coronary computed tomography angiography (CCTA), which has high diagnostic accuracy for CAD detection in patients with low or intermediate pretest probability. However, in patients with positive findings and higher pretest probability, it lacks in specificity [3]. In 2019, the European Society of Cardiology renewed their major recommendations for basic testing, diagnostics, and risk assessment. CCTA is recommended as the initial test for diagnosing CAD in symptomatic patients in whom obstructive CAD cannot be excluded by clinical assessment alone. Functional imaging for myocardial ischaemic is recommended if CCTA has shown CAD of uncertain functional significance or does not provide diagnostic value [4].

The conventional method for the diagnosis of functionally relevant myocardial ischaemic is single-photon emission computed tomography. It has been applied for decades and is widely available and commonly used in clinical practice [5]. However, rest/stress single-photon emission computed tomography imaging suffers from low temporal and spatial resolution [5, 6]. There are two x-ray-based methods for functional testing, namely invasive coronary angiography optionally in combination with fractional flow reserve measurement and cardiac dynamic perfusion computed tomography (CT) imaging. The main advantage of CT is the high spatial resolutions, which offer the possibility of anatomical diagnosis like coronary stenosis. In combination with a dynamic CT acquisition, it can offer a combined assessment of stenosis and functional parameters [7, 8]. Huber et al. [3] showed that the evaluation of dynamic CT stress perfusion images of the myocardium provides high diagnostic accuracy compared with invasive coronary angiography and fractional flow reserve measurement. Spectral or dual-energy CT with iodine density maps offers higher contrast-to-noise ratio and signal enhancement regarding contrast agent sensitivity for cardiac dynamic perfusion imaging [9]. Especially in regions with low contrast accumulation like the myocardium, improved quantification of the iodine density down to 0.5 mg/mL plays a crucial role [10, 11].

The detector coverage of most dual-energy CT systems in spectral mode is not sufficient to cover the whole myocardium during perfusion measurements within one shot, making it inapplicable for patient perfusion measurements at time of this study. Nevertheless, there are systems available with a high detector coverage of up to 16 cm that can run in sequential dual energy mode, acquiring spectral information in two consecutive heartbeats. The image-based material decomposition in sequential mode is prone to movement artifacts which leads to biases in the iodine density images. Also, because of the electrocardiographic triggering in dual-energy mode, there would be a halving of the number of available time points for the dynamic perfusion.

In this proof of principle study, we use spectral data acquired by a dual-energy CT system for cardiac dynamic perfusion, examining a dynamic three-dimensional (3D)-printed heart phantom with simulated stenosis to utilise the increased contrast-to-noise ratio of dual-energy CT for the assessment of functional parameters.

## Methods

### Phantom design

**Fig. 1:**
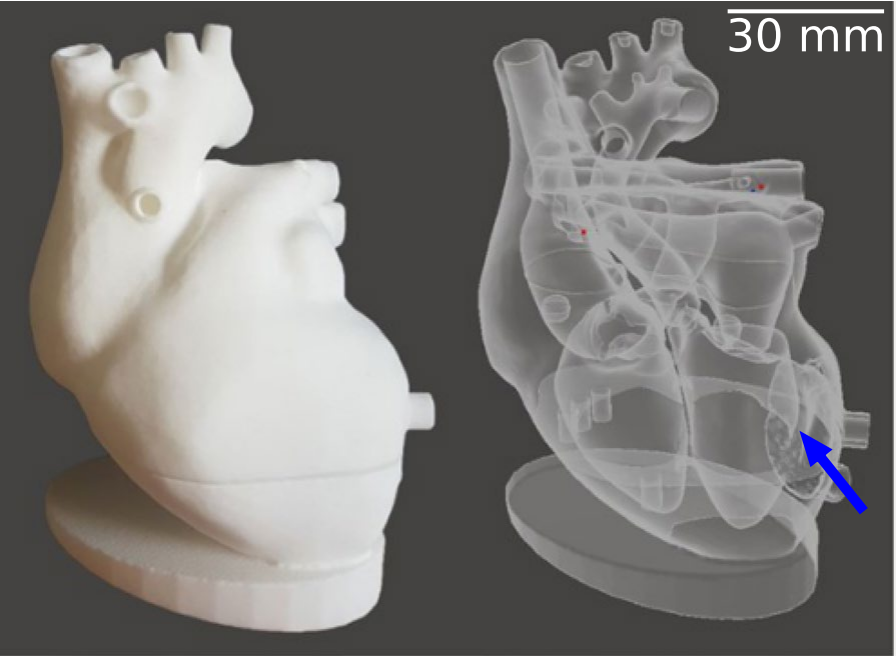
Three-dimensional (3D)-printed model is shown as photography (*left*) and 3D rendering (*right*). The phantom inflow is situated at the top left simulating the blood influx from the end-piece of the superior vena cava into the right atrium. Water is streaming through the opened tricuspid valve into the right ventricle from where the pulmonary circle is fed. The pulmonary resistance is simulated using a tube connection from the pulmonary arteries to the pulmonary veins. Passing the left atrium, left ventricle, and aortic valve, the contrast bolus splits into three outflows. The beginning of the aorta represents the blood flow of the body circuit. Two smaller outflows at the beginning of the aorta act as right coronary artery and left anterior descending artery. The reduced flow of the myocardium is simulated by a sponge-like 3D printed structure (blue arrow). An additional connection from the myocardium acts as a sink

The heart model was used to simulate the flow circulation in a human heart. The study topic could in principle be investigated in a small sponge-filled cavity with two inflows and an outflow. In this simpler form, the model would be better controllable. Nevertheless, a heart model was used to get closer to a clinically relevant situation, where also further parameters, like the arterial input function (AIF) or cavity volume, correspond more precisely to real dynamic perfusion measurements in cardiac imaging. To model the circulation dynamics within the ventricles and the heart muscle, significant simplifications were made regarding the human circulatory system. Since in our designed study only haemodynamic processes of the heart were tested, all processes before the blood inflow through the superior and inferior vena cava and after the blood outflow from the aortic arch were neglected. The blood inflow into the cardiac system was simulated by an inflow of water with variable flow velocity. The sink was modeled by two tubes exiting the left ventricle and the myocardium. The pulmonary circulation was simulated by two tubes with appropriate flow resistance. Furthermore, a sponge-like 3D-printed structure was located in the left ventricle’s heart muscle to mimic the contrast medium perfusion in the myocardium. A schematic view of the flow through the 3D-printed heart phantom is visualised in the supplementary materials. Perfusion of water and contrast agent mixture is only possible in the material-free part of this structure (active myocardial area), which is in the following also called “myocardium-like tissue.” A photographic view and a volume rendered picture of the phantom anatomy are shown in Fig. 1. A 3D rendering of the myocardium-like tissue is visualised in the supplementary materials. In this 3D model, no exchange between compartments is considered, and only a reduction of flow velocity through myocardium-like tissue is simulated, which can be motivated by a negligible permeability-surface exchange in comparison to the blood flow within the vessels [12] (Additional file 1).

For the construction of the phantom geometry, a patient CCTA scan from a 64-slice single source dual-layer CT scanner (IQon, Philips Healthcare, Best, The Netherlands) was used.^1^ The CCTA protocol was acquired in helical acquisition mode centred around the left ventricle. An acceleration voltage of 120 kVp and a current time product of 40 mAs was applied. A soft convolution kernel (B) with a pixel spacing of 0.8 mm and a slice thickness of 0.9 mm was used for reconstruction. From this scan, a true-to-scale model of the heart chambers and blood vessels was obtained using segmentation software. The segmentation has been simplified by excluding small blood vessels and complicated structures in order to enable a robust 3D model. The extensive simplifications made to the phantom make it very generic to any patient’s anatomy. Right and left ventricle wall thickness was approximately equivalent, considering that wall thickness and structure do not affect noise values, as the absorption of the 3D-printed materials plays a minor role in total absorption (compared to the absorption from the water bath). A laser sintering polyamide powder (PA2200, EOS GmbH, Munich, Germany) was used for fabrication in a commercial 3D printer (Formiga P110, EOS GmbH, Munich, Germany). The additive technology allows all components to be printed without supporting structures. PA2200 has absorption properties similar to those of the human tissues and is water-resistant [13]. In this study, it acts as a container (ventricles-like) and flow-limiting structure (myocardium-like). The “.stl” files can be made available by requesting them to the corresponding author.

Considering the strongly simplified blood supply into the myocardium-like tissue, a region supplied by left anterior descending and right coronary arteries, was simulated. These vessels are modeled using two connection tubes from the aorta directly supplying the myocardial tissue. The lumen of the right coronary artery was then reduced to 25% of the original areal cross-section. This value was determined empirically by checking the effect of a blockage on contrast agent distribution into the myocardium. The comparison of blocked (right coronary artery) and opened (left anterior descending artery) supply tubes was made within one perfusion scan and the perfusion analysis was then performed on the axial view, which intercepts the plane of the two myocardial inflow tubes. The simplified geometry of the phantom with only two supplying tubes is inherently more efficient in the middle region of the simulated myocardium closer to the coronaries than the external regions. No pulsatile flow was utilised.

### Experimental setup and protocol settings

A standard myocardial perfusion imaging protocol with a fixed tube voltage of 120 kVp and an exposure of 100 mAs per time point was used on a 64-slice single source dual-layer CT scanner with a detector coverage of 4 cm and a rotation time of 0.27 seconds (IQon, Philips Healthcare, Best, The Netherlands). To get an accurate sampling of the contrast agent flooding, 36 time points over 26.9 seconds were imaged, resulting in a total dose-length product of 1,296 mGy*cm and a CT volume dose index of 326 mGy (36 time points times 9 mGy per scan). With a thorax conversion factor 0.015 mSv/(mGy*cm), this accumulates to an effective dose of approximately 19 mSv. An axial perfusion protocol with electrocardiographic triggering at 40% of the heart rhythm was used. To simulate realistic acquisition time points, the electrocardiographic device was connected to a colleague standing in the control room, who had a heart rate of approximately 70 bpm. As only a part of the myocardial tissue was simulated in this simplified model, “full coverage” of the region of interest was achievable without shuttle mode.

The heart phantom inside a water bath, mimicking adequate human absorption, was placed in the dual-layer CT unit, and connected to the water tap via a plastic hose. A dual syringe injection system (Stellant, MEDRAD, Inc., Indianola, PA, USA) was connected to the superior vena cava by a cannula. The perfusion measurement was started a few seconds after the contrast medium injection (1.0 mL/s CA for 10 s with an iodine concentration of 400 mg/mL followed by NaCl solution). Flow parameters were adapted to produce resolvable time attenuation curves with a full width half maximum of approximately 10 s and maximum values of around 8 mg/mL or 250 HU in the AIF. Spectral raw data were reconstructed using a standard soft tissue filter kernel (type B) with an axial slice thickness of 1.0 mm, a 1.0-mm slice interval, and a pixel spacing of 0.49 pixel/mm (generated with IntelliSpace Portal 11.0, Philips Healthcare, Best, The Netherlands). A soft reconstruction kernel (type B) was used for both the conventional HU and the spectral iodine density images. The experiment was performed four times to work out the correct flux of contrast agent and water corresponding to time-attenuation curves expected in patients. Unfortunately, due to variable frame conditions like the water supply to the phantom, no inter-experimental comparison was investigated within the scope of this study.

### Postprocessing software

A trace-kinetic modeling software tool [12] was adapted for postprocessing voxel-wise time attenuation curves (TAC) using a 1-compartment model for the description of dynamic contrast-enhanced images [14]. There are mainly two reasons for using tuneable postprocessing. The clinical perfusion software, available at site, is not capable of handling spectral datasets. Also, the simplified heart model poses challenges to the clinical software, which is optimised for real heart perfusion measurements. The Python software could be adapted to these circumstances. The extracted model parameters were used to calculate quantitatively evaluable results like blood flow, blood volume, and mean transit time (MTT) maps. The AIF was measured within the “right ventricle.” Bolus-based perfusion methods typically obtain the AIF from an easily visible anatomical region, such as the left ventricle. In this case, the myocardium-like tissue, supplied from the right coronary artery and left anterior descending artery, was simulated. The myocardium-like tissue insert was situated in the left ventricle wall. No myocardial tissue was simulated within the right ventricle, making it a stable region for determining the AIF. TACs were modeled using the formula:

$$TAC=A\ast convolve\left( AIF,{e}^{\left(-\lambda \ast T\right)}\right)$$

where *T* corresponds to all measured time points.

The bound-constrained minimisation method L-BFGS-B [15–17] algorithm from the Python library SciPy was used for the fitting of the TAC. A minimum example of the implemented code is provided in the supplementary materials. To calculate the errors associated to the fitting parameters, the formula

$$\Delta {x}^i=\sqrt{\ {f}_{tol}\ast {\left({H}^{-1}\right)}_{ii}}$$

was used, where Δ*x*^*i*^ determines the error of fit parameter i (*A* and *λ* from the one compartment model), *f*_*tol*_ is the upper bound where the minimisation routine stops iterating, and *H*^−1^ is the inverse Hessian matrix.

### Classification accuracy analysis

Area under curve (AUC) at the receiver operating characteristic (ROC) analysis were computed for a binary classification of voxel values into ischaemic and nonischaemic. This was done for two regions within the myocardium-like tissue to quantify the ability of differentiating different degrees of hypoperfusion. AUC values were calculated using the Python open-source software library scikit-learn 1.0 [18]. The ROC curves were generated by a simple threshold classifier (if MTT was over the threshold, then voxel was hypoperfused). No probabilities were assigned to individual voxels. A bootstrapping analysis was applied on receiver operating characteristic curves to investigate the confidence intervals for the different AUC values (bounds percentile of 5% to 95% and number of bootstraps 1,000).

### Dose reduction

Phantom measurements were performed using a high dose equal to 9 mGy CT volume dose index for every time point. To reduce dose, several scenarios are conceivable. By reducing the tube current, the exposure per rotation can be decreased. This will lead to a decrease in the signal-to-noise ratio (SNR) in the reconstructed images. Iterative reconstruction techniques may be able to compensate for that. Within the scope of this investigation, the 1-compartment contrast kinetic modeling was tested on reduced sampling points using spectral dynamic perfusion data. Only 8 of 36 time points were utilised to calculate perfusion parameters. The sampling rate was artificially decreased from approximately 0.8 to 3.0 s for all TACs including the AIF. By following this very simple approach, a dose reduction of approximately 78% from 19 mSv to 4.3 mSv can be achieved.

## Results

### Fit accuracy of postprocessing

**Fig. 2:**
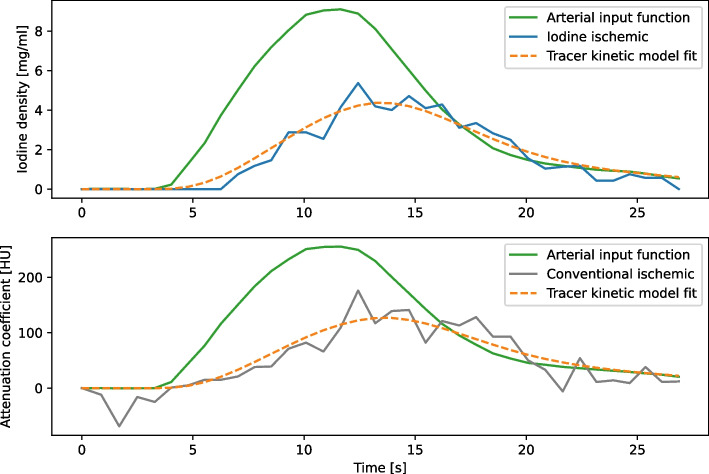
Tracer kinetic fit behaviour of spectral (top) and conventional (bottom) perfusion data in highly affected myocardial tissue. The signal-to-noise ratio (SNR) of time attenuation curves (TACs) is calculated by dividing the maximum value of the tracer kinetic fit function by the standard deviation of the error of the fit. Decreased fit parameter errors at this voxel can be calculated for the spectral perfusion measurement compared to the conventional TAC fit (42% reduction for A and 40% for λ)

The SNR in a homogenous region inside the “right ventricle” at a maximally enhanced time point increased from 7.40 to 112.02 from conventional HU to iodine density maps. Using a distinct point within the active area of the stenotic region, a decreased error on fit parameters from conventional to spectral TACs (42% reduction for *A* and 40% for *λ*) can be shown. The fit behaviour for one specific voxel within an ischaemic region is visualised in Fig. 2.

### Iodine-based perfusion maps

**Fig. 3:**
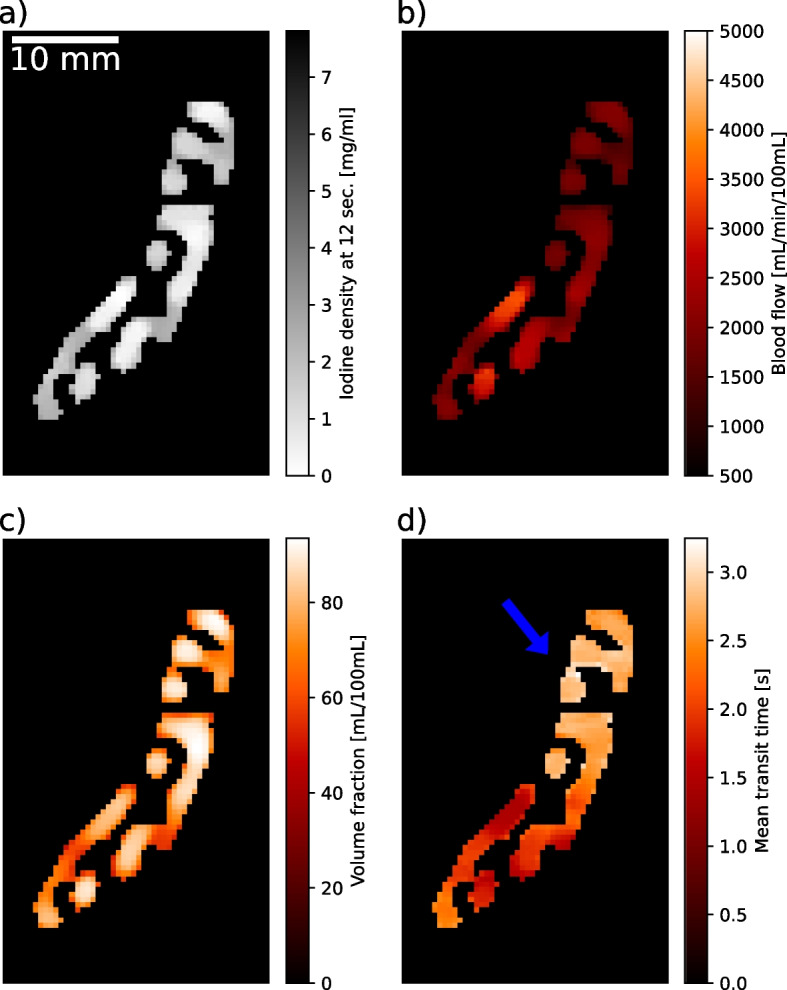
Absolute and quantitative iodine-based analysis of myocardial dynamics derived from 1-compartment fitting of iodine density-to-time attenuation curves in the active myocardium-like tissue area. The phantom’s spongy myocardium-like tissue in axial view is imaged. **a** Iodine density image at 12 s of the perfusion protocol in greyscale to distinguish it from perfusion maps. **b** Image with blood flow normalised to the tissue volume (mL/min/100 mL). **c** Image showing the volume fraction representing the compartment volume relative to the total volume of the region of interest (*e.g.*, mL/100 mL). **d** Mean transit time map. Image parts, which appear bright red in **d** are classified as haemodynamically affected myocardium-like tissue (blue arrow)

The absolute, quantitative iodine TACs used to calculate blood flow, volume fraction, and mean transit time parameter maps are depicted in Fig. 3. Three characteristic regions in all maps can be defined corresponding to regions of interest which are high, moderately, and unaffected by the simulated stenosis. The best distinction of those regions can be seen on MTT maps. The upper part of the displayed window was strongly affected by the introduced stenosis. MTTs over 2.2 s were observed in the spectral data. An unaffected region was located in the apical part of the myocardium. Here, the supplying artery (tube) was not blocked during the experimental setup. MTTs below 2.0 s were measured in this area for spectral data. The very ending of the apical region of the phantom myocardium-like tissue could be classified as moderately affected. The mean value and standard deviation for the highly affected region in MTT maps calculated from conventional and spectral data was 3.08 ± 0.44 and 2.73 ± 0.13 s, respectively.

**Fig. 4:**
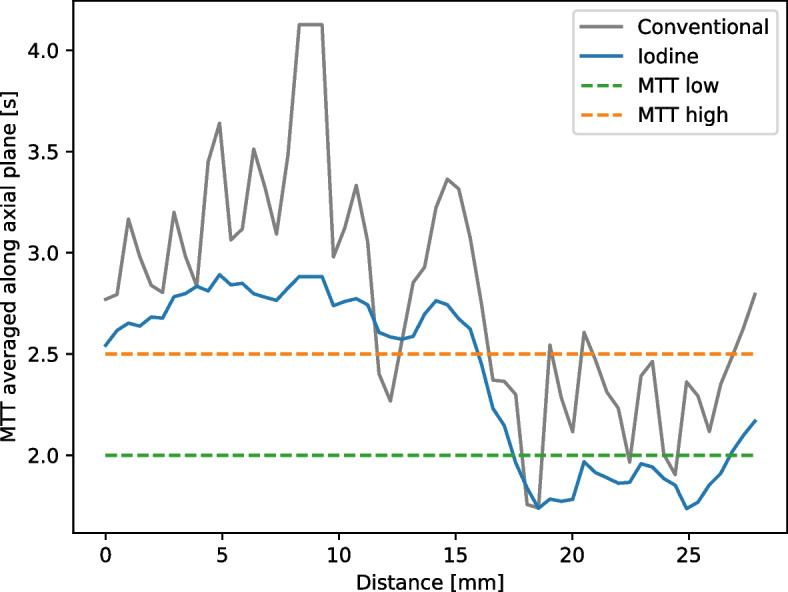
Mean transit time (MTT) line profile from top to bottom of the myocardium in axial view (in reference to Fig. 3). Voxel values from iodine and conventional measurements are averaged along one row (left to right) in axial view to reduce variance. The *x*-axis displays distance starts at zero for the top row of the myocardium-like tissue. Highly affected, unaffected, and moderately affected myocardium-like tissue from left to right. Thresholds are plotted at 2.5 and 2.0 s for the differentiation between highly and moderately affected tissue (orange-dashed line) and unaffected and moderately affected regions (green dashed line), respectively

An averaged line profile of the MTT map plotted through the myocardium-like tissue from top to bottom is presented in Fig. 4. The average was calculated over all MTT values along the *x*-axis from Fig. 3d, where MTT values were not zero. The line profile goes along the *y*-axis of Fig. 3d, plotting the averaged values. All voxel values in the active myocardium-like area along one row were averaged to get a reduced noise line profile. High variations in the conventional MTT line plot could be observed. The differentiation between the varying affected myocardium-like tissues was strongly affected by a high noise contribution in the conventional measurement. Iodine-based MTT maps showed a comparably small noise contribution (as previously reported, approximately 40% error reduction in fit parameters) with a step-like function through the differently affected tissue and continuous gradient between the plateaus, allowing to introduce thresholds of 2.5 and 2.0 s for the differentiation between highly and moderately affected tissue (orange dashed line) and unaffected and moderately affected regions (green dashed line) in spectral data, respectively. The thresholds were determined empirically from spectral MTT maps, particularly for this phantom design, and cannot be adapted to human myocardial perfusion straightforwardly. The absolute values of spectral MTT decreased as well in the unaffected as in the ischaemic area compared to conventionally calculated MTT maps, resulting from variable arterial input functions (AIFs). A more detailed investigation can be found in the supplementary materials.

### Classification accuracy analysis

**Fig. 5:**
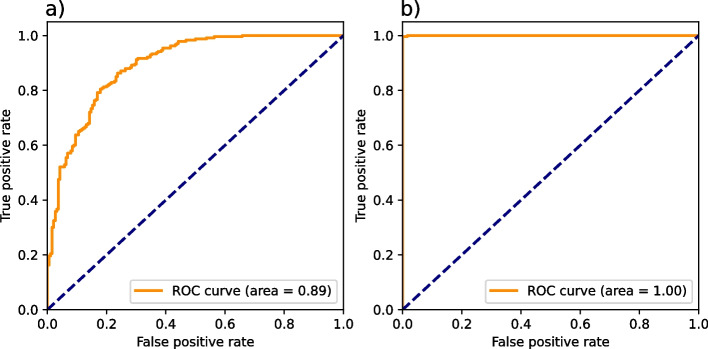
Receiver operating characteristic (ROC) analysis for conventionally (**a**) and spectrally (**b**) derived mean transit time (MTT) values. The ground truth classification of voxels to ischaemic and nonischaemic regions is based on the distance of the voxels from the blocked and non-stenotic supplying artery, respectively. The area under the curve value of 1.00 from the spectrally derived MTT ischaemia classification was achieved due to the continuous MTT decrease from ischaemic to nonischaemic regions. The blue dashed line signifies random distribution

ROC curves are displayed in Fig. 5. The ground truth values for ischaemic *versus* nonischaemic voxels are selected based on the distance of the voxels to the supplying stenotic and non-stenotic coronary artery, respectively. Voxels above the middle line (*y*-axis) of Fig. 3 are labeled as affected tissue. For the area under the curve (AUC) analysis, the highly and moderately affected region were combined and compared with the unaffected region. The AUC analysis was only performed on one *z*-slice, which intercepts both supplying tubes. The ROC curves illustrate the classification accuracy based on the MTT maps from conventionally acquired perfusion maps *versus* MTT maps from iodine perfusion measurements. The perfect AUC value of 1.00 demonstrates the continuously decreasing MTT value from ischaemic to nonischaemic regions in spectral perfusion measurements. Due to lower fit accuracies and higher fluctuations (refer to the “Fit accuracy of postprocessing” section) in conventional measurements, the voxel-wise classification accuracy was reduced to an AUC value of 0.89. A bootstrapping analysis led to following results for AUC values and 95% confidence intervals:

- classification with conventional MTT maps: 0.893 [0.868–0.917];
- classification with spectral MTT maps: 1.000 [1.000–1.000];
- classification with spectral reduced dose MTT maps: 0.961 [0.946–0.975].

### Dose reduction

**Fig. 6:**
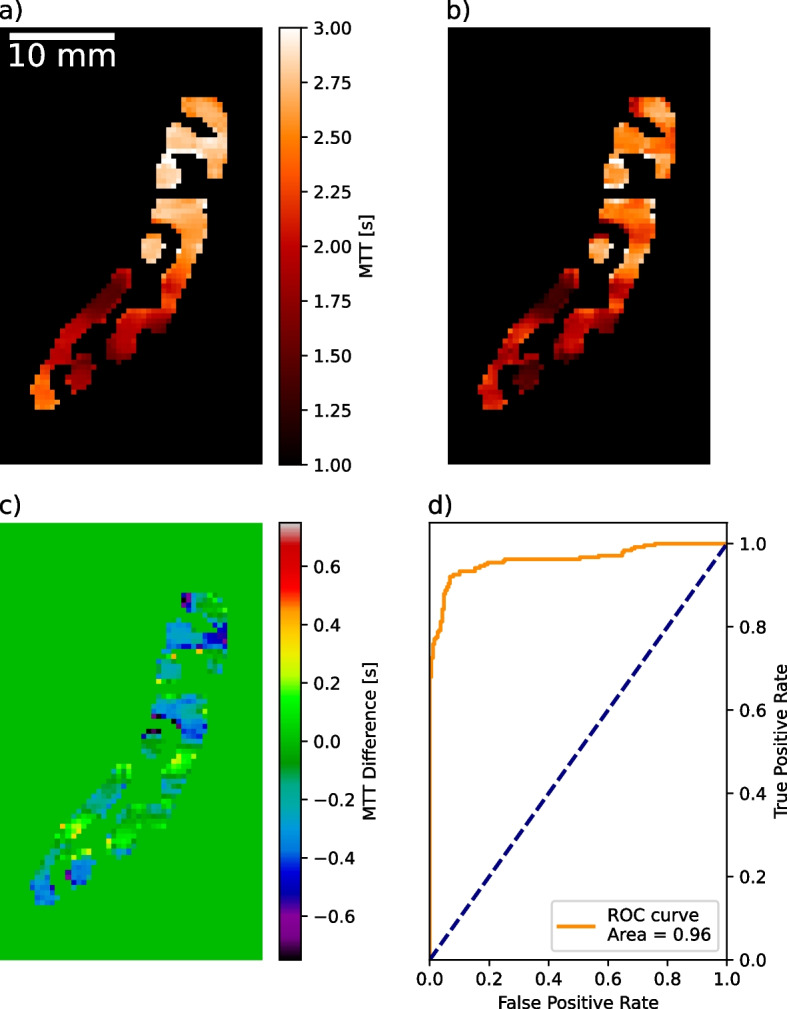
Comparison of normal dose iodine derived mean transit time (MTT) map (**a**) with simulated reduced dose dynamic perfusion acquisition (**b**). **c** differential image of normal and reduced dose measurements. **d** Receiver operating characteristic analysis for classification characterisation of stenotic regions using the reduced dose data. Deviations within the MTT map of the ischaemic and nonischaemic regions increase when applying the contrast agent kinetic model to the reduced number of time points. Nevertheless, a differentiation is achievable using the reduced dose with an area under the curve of 0.96

Classification accuracy for the differentiation of ischaemic from nonischaemic regions was tested on MTT maps calculated from normal dose and reduced dose spectral TACs. The AUC value decreases to 0.96 (Fig. 6d), because of the reduced fit accuracy from the contrast kinetic 1-compartment model on 8 time points (63% smaller error in parameter *A* from normal sampling; *λ* with no difference in fit error). Relative deviation in averaged blood flow values calculated from reduced sampling TACs was 9.3%. When comparing MTT maps visually (Fig. 6a, b) increased inhomogeneities within the different regions could be observed. Not every voxel could be classified correctly based on threshold values; however, the visual impression still allowed to determine the site of the ischaemic region (Fig. 6b, top part).

## Discussion

Validation measurements for dynamic processes using CT require the use of special phantom models. To demonstrate the detectability of stenoses with a dual-energy CT scanners, the determination of haemodynamic parameters by dual-energy CT dynamic myocardial perfusion imaging was performed. An anatomical heart model was designed and produced using 3D-printing techniques. The dynamic heart phantom could be flooded with water and contrast medium. The flow through the heart chambers and into the myocardium was imitated.

An increased SNR from conventional to spectral perfusion data (from 7.40 to 112.02) lead to a fitting process with smaller errors on fitting parameters and derived perfusion parameters using a 1-compartment model. Because the material decomposition is based on the photoelectric effect and Compton effect-based images, the anticorrelated noise can be modeled and removed from the image [19, 20]. The material decomposition provides a low-noise image with a relatively high signal. According to Scherer et al. [9], iodine density maps show the highest signal enhancement and contrast-to-noise ratio within several spectral parameter maps. Also, this inherently reflects the contrast agent flooding and washout behaviour and generates a comprehensive baseline at 0 mg/mL.

Our spectral perfusion data showed a step-like function through the differently affected tissue, while the noise contribution in conventionally derived data forbids this voxel-wise analysis indicated by high fluctuations in the line profile. For conventional perfusion data, Gaussian smoothing in plane or an increased slice thickness [7] for averaging in *z* direction must be applied. The smoothing operation will decrease the spatial resolution in all contrast kinetic fit derived maps.

The reason for the reduced flow in the very ending of the apical region of the phantom’s myocardium is due to the haemodynamic design of the myocardium-like tissue, having only two supplying tubes. The moderately affected outer region was therefore not considered in the data analysis. A dose reduction of approximately 78% from 19 mSv to 4.3 mSv was achieved by reduced sampling of time points. Visual differentiation between ischaemic and nonischaemic regions was still possible. By reducing the current time product and using iterative reconstruction techniques to keep the SNR at a constant level, we expect to be able to reduce the radiation dose to less than 2.0 mSv, being well below all reported dose values for dynamic CT myocardial perfusion studies as reported by Varga-Szemes et al. [8] from 9.4 to 18.8 mSv.

In this study, we used a dual-layer CT system. A drawback of this technology is the detector coverage of only 4 cm, which decreases the field of view in the *z* direction, limiting the assessment of the full myocardium within one shot. However, one can expect that spectral CT technologies improve rapidly towards larger detector coverage to image the whole myocardium without additional table movement. Other spectral acquisition technology like fast emerging photon-counting CT is also conceivable. This technology can acquire images at very high spatial resolution without electronic noise and with improved tissue contrast [21]. Simultaneous material decomposition of two or three contrast agents may develop the possibility of simultaneous acquisition within the time period of flooding, saturation, and late enhancement. This approach could be used to decrease dose or increase the sampling rate without having to increase the number of CT acquisitions.

There are some other publications regarding the use of phantom models in cardiac imaging. Boltz et al. [22] constructed an anthropomorphic beating heart phantom to analyse cardiac CCTA protocols. The phantom was anatomically and functionally designed to be very close to a real-world situation. For example, the beating and corresponding electrocardiographic signals can be modeled to investigate motion artifacts and perform stent imaging. Also, the chambers can be flooded with a mixture of water and contrast agent. Nevertheless, this phantom design was selected to investigate anatomically relevant information. Dynamic perfusion within the myocardium (*e.g.*, time attenuation curves) cannot be simulated using this approach. On the other hand, Chiribiri et al. [23] built a perfusion phantom that simulates myocardial first-pass magnetic resonance perfusion. This approach could easily be adapted to CT perfusion by using iodinated contrast agents instead of gadolinium-based contrast agents. The phantom establishes a very controlled and reproducible environment for modeling dynamic perfusion. In comparison to our approach, it is not able to reproduce the correct anatomically relevant structures of a human heart. Beam hardening artifacts from highly absorbing contrasted heart chambers would not be represented by using the approach by Chiribiri et al. [19]. Also, the phantom design chosen in this approach is static and cannot simulate movement artifacts from the heart. Heart movement is a source of potential bias in real patient measurements. It can be superimposed by breathing. The static 3D-printed structure neglects these issues. An elastic image registration-based method to improve the characterisation of CT-based estimates of myocardial perfusion can be applied to reduce these artifacts [24].

The phantom design with two connection tubes directly supplying the myocardium-like tissue allows better flow control in our model since the 3D-printed parts are static and no luminal control/stenosis would be possible. The lumen of the right coronary artery was then reduced to 25% of the original areal cross-section via clamping. This resulted in a degree of uncertainty being a limitation, which we will address in future work. Integrating a pulsatile flow and pressure dependent elastic vessels in our experiments will increase the accuracy and the performance of the quantitative perfusion analysis towards more realistic data. For our proof-of-principle experiments to assess if a stenosis and the consequent reduced myocardial perfusion can be analysed, we think that this single degree of perfusion is sufficient.

The assessment of *in vivo* patient data is crucial for further investigations towards spectral dynamic myocardial perfusion imaging. A recent study [7] investigated dynamic myocardial perfusion CT in nine centres around the world finding an incremental diagnostic value compared to CCTA as a standalone modality. Especially, a higher specificity (72% for CCTA, 89% for CCTA plus perfusion imaging) offers the opportunity to reduce unnecessary cardiac interventions after anatomical assessment alone. Spectral acquisition may be the key technology to overcome the limitation of low contrast to noise ratio in CT [25]. Fahmi et al. [26] showed increased inhomogeneities (dark band on the myocardium) in conventional 120-kVp pig images resulting from beam hardening artifacts from highly absorbing ventricles. These artifacts could be avoided by using 70-keV virtual monoenergetic images leading to a more reliable assessment of nonischaemic defects.

A full CT analysis of the heart including calcium scoring using CCTA and a dynamic myocardial perfusion could be performed within 10 minutes. This workflow may provide a comprehensive, one-stop, noninvasive and superior method for the evaluation of CAD and myocardial perfusion.

## Supplementary Information

**Additional file 1.** Electronic supplementary material.

## Data Availability

The datasets used and/or analysed during the current study are available from the corresponding author on reasonable request.

## Abbreviations

3D: Three-dimensional
AIF: Arterial input function
AUC: Area under the curve
CAD: Coronary artery disease
CCTA: Coronary computed tomography angiography
CT: Computed tomography
MTT: Mean transit time
ROC: Receiver operating characteristic
SNR: Signal-to-noise ratio
TAC: Time attenuation curve

## Footnotes

1 A scan of a patient was used. The extensive simplifications made to the phantom make it very generic to any patient’s anatomy and impossible to draw conclusions about the patient’s identity. The CCTA was only important for the true-to-scale model.
