# Supplementary material for: Absolute iodine concentration for dynamic perfusion imaging of the myocardium: improved detection of poststenotic ischaemic in a 3D-printed dynamic heart phantom
Source: Eur Radiol Exp. 2022 Oct 31;6:51. doi: 10.1186/s41747-022-00304-x (PMC9618471; doi:10.1186/s41747-022-00304-x)
Supplement: Supplementary file 1 — Additional file 1. Electronic supplementary material. [file 41747_2022_304_MOESM1_ESM.pdf]

ELECTRONIC SUPPLEMENTARY MATERIAL

Absolute iodine concentration for dynamic perfusion imaging of the myocardium: improved detection of poststenotic ischaemic in a 3D-printed dynamic heart phantom

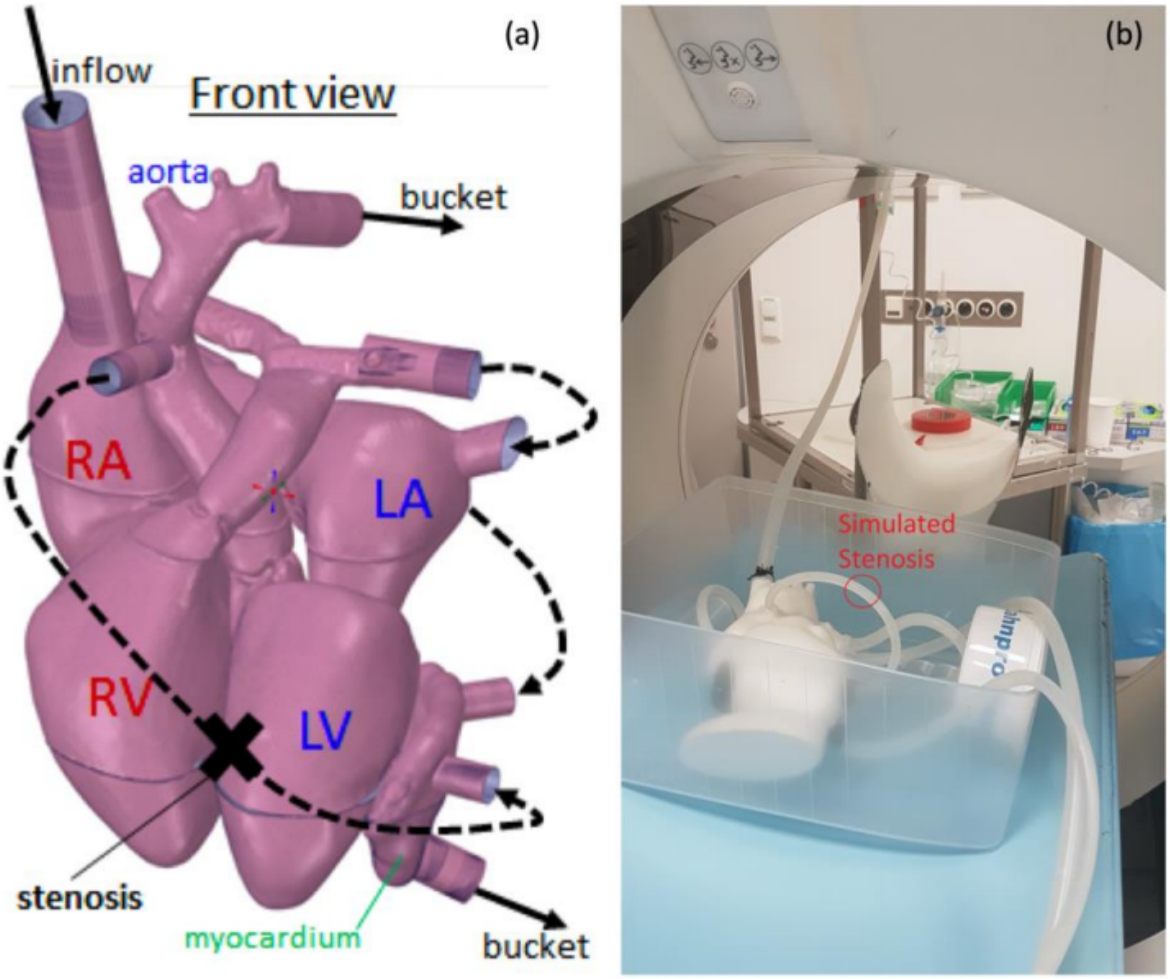

Figure 1

(a) Inflow, outflow (bucket) and connecting arrows indicate the location of the connecting tubes. Right atrium (RA), right ventricle (RV), left atrium (LA) and left ventricle (LV). (b) A picture of the heart model within the gantry of the spectral CT system. During the performance of the dynamic scan, the container is filled with water.

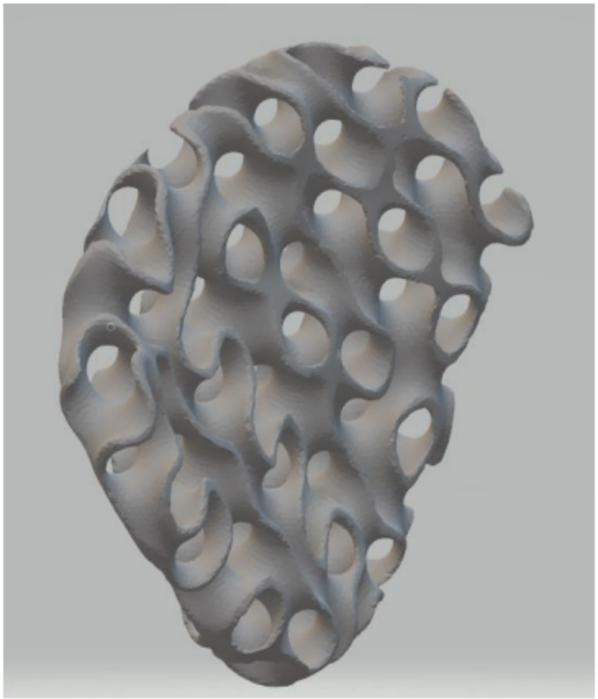

Figure 2

3D rendering of the myocardial structure simulating the reduced flow in the myocardial region.

The absolute values of spectral MTT are decreased as well in the unaffected as in the ischemic area compared to conventionally calculated MTT maps, resulting from variable arterial input functions (AIFs). This is a general problem of tracer kinetic modeling using the arterial input function as a “fit reference”. The AIF must be selected manually by drawing a ROI within the suppling vessel. In our experiment, this ROI was the same for iodine data as well as conventional data. The selection still leads to minor variations in the time attenuation curves (TACs). This can be visualized (figure 3) by plotting the normalized AIFs of iodine and HU data. Also, in figure 3 the normalized TACs of an ischemic voxel and their corresponding fits are plotted. The fit behavior looks very similar, but the fit parameters and therefore the derived physical parameters like MTT deviate because the initial AIFs differ. In the current example, an MTT value of 2.89 and 3.17 seconds was calculated for the iodine and conventional fit respectively.

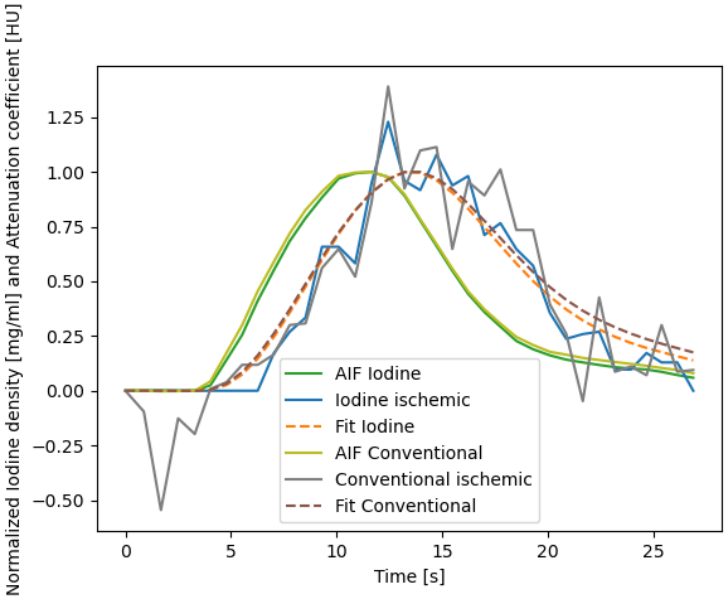

Figure 3

Normalized arterial input function from iodine and HU data and corresponding TAC fits of ischemic voxels are shown. Please consider, that all curves are normalized to have a maximum value of 1.00.

Minimum example of one compartment model fit:

```
import numpy as np
from scipy.optimize import minimize
import matplotlib.pyplot as plt

# sample AIF (arterial input function) using iodine density [mg/ml] map
aif = np.array([0.  , 0.01, 0.01, 0.  , 0.01, 0.22, 1.29, 2.32, 3.74, 5.02, 6.22,
       7.18, 8.03, 8.82, 9.04, 9.1 , 8.88, 8.11, 7.08, 6.02, 4.98, 4.03,
       3.27, 2.66, 2.07, 1.72, 1.47, 1.29, 1.18, 1.07, 0.98, 0.92, 0.88,
       0.79, 0.65, 0.54])

# sample voxel inside simulated myocardium [mg/ml]
myo_iod = np.array([0.  , 0.  , 0.  , 0.  , 0.  , 0.  , 0.  , 0.  , 0.  , 0.75, 1.18,
       1.46, 2.87, 2.87, 2.54, 4.14, 5.36, 4.19, 4.  , 4.71, 4.09, 4.28,
       3.11, 3.34, 2.82, 2.49, 1.55, 1.04, 1.13, 1.18, 0.42, 0.42, 0.75,
       0.56, 0.56, 0.])

# time points of dynamic perfusion measurment [s]
times = np.array([ 0. ,  0.9,  1.7,  2.5,  3.3,  4. ,  4.8,  5.5,  6.3,  7. ,  7.8,
        8.5,  9.3, 10.1, 10.9, 11.6, 12.4, 13.2, 13.9, 14.7, 15.5, 16.2,
       17. , 17.7, 18.5, 19.3, 20.1, 20.9, 21.6, 22.4, 23.2, 23.9, 24.7,
       25.4, 26.1, 26.9])

curve = myo_iod

# convolution of aif using an exponential function with fit parameter lambda (lam)
def convolution(times, lam, aif):
    expon = np.exp(-lam * times)
    y = np.convolve(aif, expon, mode='full')
    # scaling down by a factor dt
    y = y * (times[1] - times[0])
    # first half of convolution
    half = int(len(y) / 2 + 1)
    y = y[0:half]
    return y

# Scaling factor + convolution
def modelfunction(parameters, times, aif):
    modelcurve = parameters[0] * convolution(times, parameters[1], aif)
    return modelcurve

# wrapper to minimize modelfunction
def min_wrapper(parameters, *args): # args = (curve, times, aif)
```

```
    diff = args[0] - modelfunction(parameters, args[1], args[2])
    return np.sum(diff ** 2)


def fit(curve, times, aif):
    startparameters = np.zeros(2)
    bounds = [(0, None), (0, None)]
    method = 'L-BFGS-B'
    fit_results = minimize(min_wrapper, startparameters, args=(curve, times, aif),
method=method, bounds=bounds)
    fitparameters = fit_results.x
    fit = modelfunction(fitparameters, times, aif)
    return fitparameters, fit

fitparameters, fit = fit(curve, times, aif)

# get physical parameters
def phys_par(fitparameters):
    # fit parameters to the 'physiological' model
    F = fitparameters[0] * 6000. # Blood flow
    v = fitparameters[0] / fitparameters[1] * 100 # blood volume
    mtt = 1 / fitparameters[1] # mean transit times
    return F, v, mtt

# convert the fit parameters back to physiological parameters
F, v, mtt = phys_par(fitparameters)
print("Physics parameters:")
print("Flow: ", F, "Volume: ", v, "Mean transit time: ", mtt)

plt.figure()
plt.plot(times, aif, label='AIF')
plt.plot(times, curve, label='Curve')
plt.plot(times, fit, label="Curve fit")
plt.ylabel('Iodine density [mg/ml]')
plt.xlabel('Time [s]')
plt.legend()
plt.show(block=False)
```
